# Coronary plaque burden as a determinant of cardiovascular outcomes in patients undergoing percutaneous coronary intervention versus coronary artery bypass grafting: The Western Denmark Heart Registry

**DOI:** 10.1016/j.ajpc.2026.101429

**Published:** 2026-02-18

**Authors:** Martin Bødtker Mortensen, Jesper Møller Jensen, Hans Erik Bøtker, Michael Maeng, Kevin Olesen, Mariann Tang, Niels Peter Rønnow Sand, Erik Grove, Kristian Kragholm, Lars Lyhne Knudsen, Martin Busk, Kristian Øvrehus, Michael J. Blaha, Patrick Serruys, Henrik Toft Sørensen, Bjarne Linde Nørgaard

**Affiliations:** a Department of Cardiology, Aarhus University Hospital, Aarhus, Denmark; b Johns Hopkins Ciccarone Center for the Prevention of Cardiovascular Disease, Johns Hopkins University School of Medicine, Baltimore, Maryland, United States; c Department of Cardiothoracic surgery, Aarhus University Hospital, Aarhus, Denmark; d Department of Cardiology, Regional Hospital Esbjerg, Esbjerg, Denmark; e Department of Cardiology, Aalborg University Hospital, Aalborg, Denmark; f Department of Internal Medicine, Regional Hospital Randers, Randers, Denmark; g Department of Cardiology, Sygehus Lillebælt, Vejle, Denmark; h Department of Cardiology, Odense University Hospital, Odense, Denmark; i Department of Cardiology, University of Galway, Ireland; j Department of Clinical Epidemiology, Aarhus University Hospital, Aarhus, Denmark

**Keywords:** CABG, PCI, Plaque burden, Coronary artery calcification

## Abstract

**Aims:**

Benefits of coronary artery bypass grafting (CABG) versus percutaneous coronary intervention (PCI) for patients with stable coronary artery disease (CAD) may be determined by total plaque burden alongside number of stenoses. We aimed to determine whether coronary artery calcification (CAC), reflecting overall plaque burden, predicts CABG versus PCI benefits.

**Methods:**

Among 85,512 symptomatic patients undergoing computed tomography angiography, we conducted propensity-matched analyses. CABG-treated patients (n = 1,479) were 1:1:1 matched with PCI-treated patients and non-obstructive CAD patients. Cox proportional hazard models assessed risk for the primary endpoint (death, myocardial infarction, stroke), stratified by CAC<300, CAC 300-1000, and CAC>1000.

**Results:**

Over 5.3 years, 710 primary endpoints occurred. CABG patients had higher prevalence of three-vessel obstructive CAD compared to PCI-treated patients (30 %vs.18 %). Event rates rose with increasing CAC scores; CAC<300 indicated low risk, CAC>1000 indicated very-high risk. Patients with non-obstructive CAD had similar risk to PCI-revascularized patients across CAC groups. Compared to PCI, risk for the primary endpoint were lower at higher plaque burden in CABG-treated patients with hazard ratios of 1.12 (95 %CI 0.70-1.80), 0.82 (95 %CI 0.55-1.20), and 0.75 (95 %CI 0.58-0.94) for CAC<300, CAC 300-1000, and CAC>1000, respectively. The lower CABG-associated risk was driven by reduced myocardial infarction risk (HR 0.44 (95 %CI 0.26-0.73)).

**Conclusions:**

Among patients with high, but not low, coronary plaque burden, CABG yielded lower long-term risk than PCI, despite more multivessel obstructive CAD. This suggest that the benefit of CABG over PCI for patients with extensive CAD can, at least partly, be attributed to the bypassing of a larger plaque burden.

## Graphical abstract

Among patients with high, but not low, coronary plaque burden as assessed by coronary artery calcification, revascularization with coronary artery bypass grafting is associated with lower long-term risk than percutaneous coronary intervention despite higher prevalence of multivessel obstructive disease.

**Figure:**
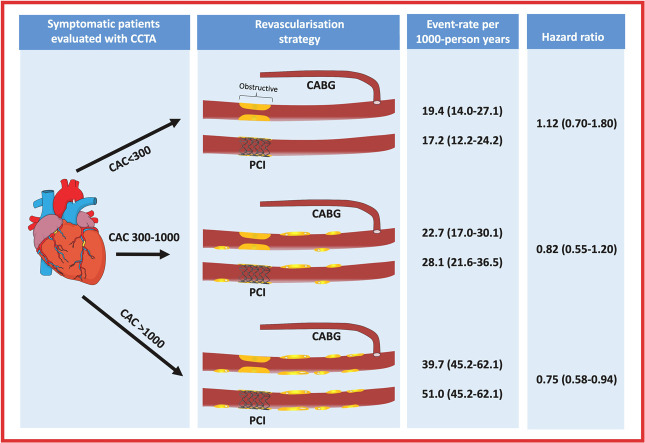

## 1 Introduction

Patients with obstructive coronary artery disease (CAD) are at high risk for future cardiovascular events [[1], [2], [3]]. However, large clinical trials have not consistently shown a reduction in hard cardiovascular endpoints with percutaneous coronary intervention (PCI) compared to medical therapy alone, casting doubt on the role of obstructive plaques in mediating the high risk [[4], [5], [6], [7]]. Conversely, coronary artery bypass grafting (CABG) has demonstrated a significant decrease in cardiovascular risk as compared to PCI, particularly in cases of multivessel CAD, attributed to a substantial reduction in coronary events such as myocardial infarctions [[8], [9], [10], [11]]. Two recent large observational studies suggest a greater benefit of CABG over PCI in patients with heart failure and non-ST elevation myocardial infarction, yet they provide no explanation for these findings [12,13]. Unlike PCI, which targets obstructive plaques exclusively, CABG addresses both obstructive and non-obstructive plaques, allowing blood flow to bypass the majority of atherosclerotic lesions. Given that non-obstructive plaques outnumber obstructive plaques, they are responsible for the majority of subsequent myocardial infarctions. Therefore, the observed advantages of CABG over PCI in patients with multivessel CAD may arise from bypassing a larger burden of plaques, encompassing both obstructive and non-obstructive types [14]. If this hypothesis is true, CABG should be expected to provide greatest benefit to patients with a high overall coronary plaque burden who are known to be at high cardiovascular risk, and provide lesser benefits to those with a low overall plaque burden, representing low cardiovascular risk, regardless of the number of vessels affected by obstructive plaques.

In the present study based on the Western Denmark Heart Registry (WDHR), we aimed to determine if the extent of coronary plaque burden, assessed by coronary artery calcification (CAC), predicts the benefits of revascularization with CABG vs. PCI in patients with stable CAD. CAC is a well-established, validated and easily measured surrogate marker of total coronary plaque burden [15,16].

## 2 Methods

### 2.1 Study cohort

The study is based on the WDHR including 85,512 consecutive patients without known CAD who underwent computed tomography angiography (CCTA) to evaluate CAD-related symptoms in western Denmark between 2008-2021. The WDHR is a semi-national, multicenter based registry that maintains longitudinal records of detailed patient and cardiac procedure data [[17], [18], [19]]. All centers doing CCTA and other cardiac diagnostics and interventions in Western Denmark report to the WDHR. In Denmark four university centers perform both PCI and CABG of which three centers (Aarhus University Hospital, Odense University Hospital, and Aalborg University Hospital) report to the WDHR. No centers in the WDHR region perform only PCI or only CABG. Pre-specified performance standards set by the Danish Heart Registry are met by all centers reporting to the WDHR [20]. The reliability and accuracy of this data source have been audited and validated [20]. Invasive coronary angiography, PCI and CABG were performed according to standard practice. For the present study, we included patients who underwent revascularization with PCI or CABG within 180 days of CCTA. The 180-day timeframe was chosen as it captures the vast majority of patients who are refereed for invasive angiography and revascularization because of the CCTA result (**Supplementary Figure 1**). Patients were followed for events from revascularization day to end of follow-up.

The Danish National Health Service provides universal tax-supported healthcare, ensuring complete financial coverage for all citizens. Exclusion criteria were known coronary artery disease (prior myocardial infarction or coronary revascularization). Each Danish citizen is assigned a Civil Personal Registration (CPR) number at birth, enabling accurate linkage of information across the medical and administrative registries employed in this study [[21], [22], [23]].

The study was approved by the Danish Data Protection Agency (record number: 1-16-02-36-21) with a waiver for individual informed consent by the regional ethics committee

### 2.2 Angiography acquisition and coronary atherosclerosis definition

All CCTA examinations were completed according to the Society of Cardiovascular Computed Tomography best practice guidelines for CCTA image acquisition [24]. Before CCTA, a non-contrast scan was acquired to obtain the Agatston coronary artery calcification (CAC) score. Patients were categorized into three groups based on the CAC score: 1) CAC<300, CAC 300-1000 and CAC >1000. CCTA defined "nonobstructive CAD" was defined as the presence of any plaque with <50 % luminal stenosis, while "obstructive CAD" was defined as the presence of plaque with >50 % stenosis. Patients were divided further according to the presence of 1 or 2, 3 or left main (LM) obstructive CAD.

### 2.3 Comorbidity assessment

Based on diagnosis codes, a Charlson Comorbidity Index (CCI) score was computed for each patient (**Supplementary Table 1**) and comorbidity was categorized as low (CCI score = 0), moderate (CCI score = 1) or severe (CCI score≥2) as previously described [[15], [16], [17],^23^].

### 2.4 Primary endpoint and adverse outcomes

The primary endpoint was a composite of all-cause death, myocardial infarction and stroke occurring until December 31, 2021. Periprocedural events (ie. periprocedural myocardial infarctions) were included in the analyses. Adverse outcomes were a diagnosis of atrial fibrillation, heart failure or bleeding events occuring after revascularization. The study outcomes were identified through linkage among national registries covering all Danish hospitals, including the Danish National Patient Register which holds records on 99.4 % of all in-patient and out-patient contacts to Danish hospitals according to the International Classification of Diseases codes, 10^th^ revision [[18], [19], [20]]. Data on prescribed medications were retrieved from the Danish National Health Service Prescription Database.

### 2.5 Statistical analyses

Baseline characteristics were presented as proportions for categorical variables and as medians with interquartile range (IQR) for continuous variables.

To account for differences in baseline characteristics among patients with non-obstructive CAD, patients with obstructive CAD treated with PCI, and patients with obstructive CAD treated with CABG, propensity score matched cohorts were created using PSMATCH2 with 1:1:1 nearest matching. Matching variables included age, sex, smoking status, hypertension, diabetes, level of Charlson Comorbidity Index (CCI) score, and statin use at baseline. Matching was performed separately for patients with CAC<300, CAC 300-1000, and CAC>1000.

The event rate per 1000 person-years with 95 % confidence intervals was calculated for the three patient groups across the three CAC groups: CAC<300, CAC 300-1000, and CAC>1000.

Cox proportional hazard models were used to assess the independent association between higher CAC scores and the risk for the primary endpoint and its individual components in each of the three patient groups. The models were adjusted for age, sex, smoking status, hypertension, diabetes, CCI levels, statin use and aspirin use.

Furthermore, Cox proportional hazard models analyzing time to first event were used to compare the risk for the primary endpoint and its individual components between patients undergoing CABG and PCI. The proportional hazard assumption was assessed using Schoenfeld residuals and was met for all models when excluding procedure-related events. Kaplan-Meier plots were created to visualize the results.

Given the inherent limitations of the observational study design, which makes it difficult to completely eliminate uncontrolled confounding, we performed sensitivity analyses to evaluate the risk of an endpoint that is unlikely to be influenced by the choice of revascularization procedure when taking into account the amount of CAC. In particular, we examined the risk of peripheral artery disease (PAD). If the propensity score matching was effective, we would expect the risk of PAD to be comparable between the two groups over the course of follow-up. Furthermore, in another sensitivity analysis aimed at further substantiating that plaque burden plays a more important role for risk than presence of stenosis, we re-analyses results by replacing the patients with non-obstructive CAD that did not undergo revascularization with patients who were classified as obstructive CAD but did not undergo revascularization, that is, patients with untreated stenoses. Finally, to assess the robustness of the results, the data were re-analyzed without creating propensity score matched cohorts. The risk for the primary endpoint after CABG versus PCI in the total population was evaluated using Cox proportional hazard models, adjusting for age, sex, smoking status, hypertension, diabetes, CCI score, and statin use. These analyses were stratified by CAC<300, CAC 300-1000, and CAC>1000.

All statistical analyses were performed using STATA17.0 (Stata Corp.,College Station,Texas).

## 3 Results

The study included 85,512 patients who underwent CCTA for evaluation of symptoms suggestive of CAD. Within 180 days, 4,942 patients were revascularized with PCI, and 1,479 patients had CABG, representing over 80 % of all procedures during follow-up. In patients undergoing PCI, CAC was <300 and >1000 in 47 % and 23 % of patients, respectively, as compared to 24 % and 47 % in patients undergoing CABG (**Supplementary Figure 2**). Prior to matching, CABG patients were older, predominantly male, had higher prevalence of hypertension and diabetes, and higher CAC scores than PCI patients (**Supplementary Table 2**). After 1:1:1 propensity matching, baseline characteristics were similar among patients who 1) had non-obstructive CAD and did not undergo revascularization (n = 1,479), 2) underwent PCI (n = 1,479), and 3) underwent CABG (n = 1,479) although the prevalence of CCTA-defined three-vessel obstructive CAD were higher among patients undergoing CABG compared to those who underwent PCI or had non-obstructive CAD (33 % vs 18 % vs. 0 %) (Table 1 and **Supplementary Tables 3-5**). Post-CCTA use of statin and aspirin was high in all three groups **(Supplementary Table 6).**

**Table 1:** Baseline characteristics of the 1:1:1 propensity score matched patients from the Western Denmark Heart Registry included in the study.

Characteristics	CABG	Matched PCI	Matched non-obstructive CAD without revascularisation*	p-values
Participants, n	1479	1479	1479	
Age, mean (years) Current smoking, %	65 (58-71) 18	65 (59-71) 18	65 (58-71) 81	P=0.10 P=0.85
Male (%)	81	82	81	P=0.57
Hypertension (%)	53	53	53	P=0.80
Diabetes (%) Previous stroke (%)	12 5	11 5	12 5	P=0.12 P=0.41
Body Mass Index, median, IQR Comorbidity level^A^ (%) None Low Moderate Severe	26.8 (24.5-29.4)) 57 21 11 11	27.1 (24.7-29.7) 59 20 9 12	27.0 (24.5-30.1) 58 20 10 11	p=0.10 p=0.65
CAC (median, IQR) 3-vessel disease**, % Left main disease***, % Pre-CTA Statin use^B^, % Pre-CTA Aspirin use^C^, %	900 (314-1870) 33 9 8 9	878 (304-1654) 18 3 6 11	857 (304-1760) 4 1 7 10	P=0.93 P<0.001 p<0.001 p=0.67 p=0.01

Data are n (%), median (IQR), or n.

A Levels of comorbidity were based on Charlston Comorbidity Index scores of 0 (low), 1 (moderate), and ≥2 (severe).

B Defined as a statin prescription redeemed >180 days before CCTA.

C Defined as an aspirin prescriptions redeemed >180 days before CCTA. *Patients with non-obstructive coronary artery disease and did not undergo revascularization within 180 days of CCTA **CCTA-defined 3-vessel obstructive coronary artery disease ***CCTA-defined left main disease

Over a median 5.3-year follow-up, the matched cohort had 710 primary endpoints, including 431 deaths, 188 myocardial infarctions, and 165 ischemic strokes. No patients were lost to follow-up.

### 3.1 Coronary artery calcium burden and risk for the primary outcome

**Fig. 1:**
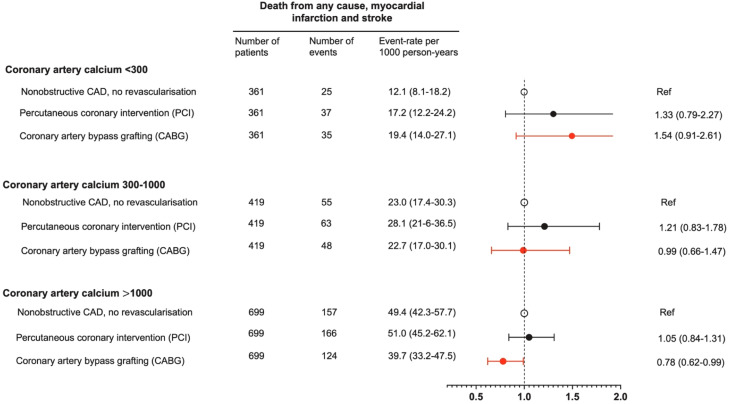
Risk for the primary endpoint in patients with non-obstructive coronary artery disease and in patients with obstructive coronary artery disease revascularized with percutaneous coronary intervention or coronary artery bypass grafting stratified by coronary artery calcification burden.

**Fig. 2:**
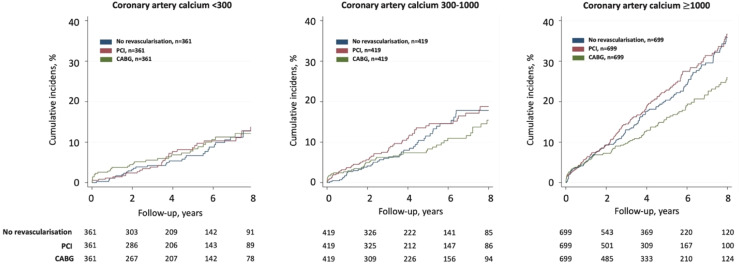
Cumulative incidence curves of the primary endpoint in patients with non-obstructive coronary artery disease and in patients with obstructive coronary artery disease revascularized with percutaneous coronary intervention or coronary artery bypass grafting stratified by coronary artery calcification burden.

Event rates per 1000 person-years for the primary endpoint increased with higher CAC scores in all three patient groups (Fig. 1, Fig. 2). The increase in event-rates with higher CAC scores was most pronounced in patients with non-obstructive CAD and patients with obstructive CAD treated with PCI with multivariable adjusted HRs of 2.72 (95 % CI 1.72-4.32) and 2.45 (95 % CI1.57-3.51), respectively, for CAC>1000 vs. CAC<300 (**Supplementary Table 7**). The association between higher CAC scores and the primary endpoint was weaker for CABG patients (HR 1.26, 95 % CI 0.84-1.88), primarily due to the lack of association with future myocardial infarctions.

Across the CAC groups, there were no significant difference in risk for the primary endpoint in patients with non-obstructive CAD compared to patients being revascularized with PCI (Fig. 1, Fig. 2). In contrast, among patients with CAC>1000, revascularization with CABG was associated with a lower risk for the primary endpoint compared to patients with non-obstructive CAD that were not revascularized, with a HR of 0.78 (95 % CI 0.62-0.99) (Fig. 1, Fig. 2).

### 3.2 Outcomes after revascularization with PCI versus CABG according to coronary artery calcium burden

**Fig. 3:**
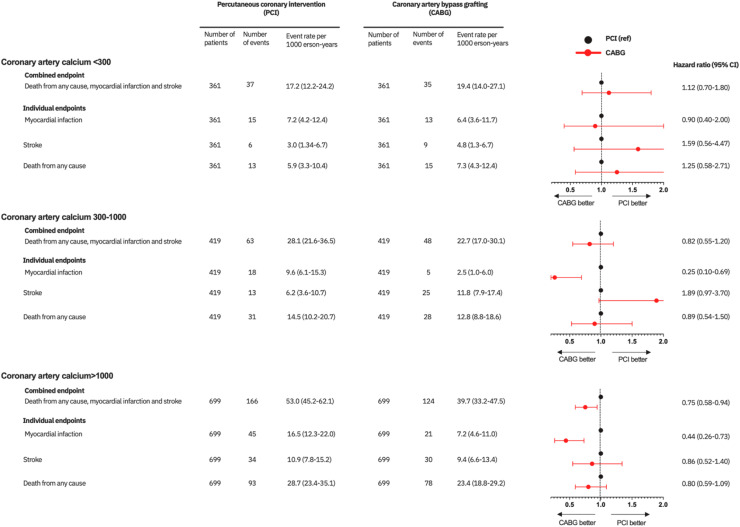
Risk for the primary endpoint and its individual components in patients revascularized with percutaneous coronary intervention compared to coronary artery bypass grafting stratified by coronary artery calcification burden.

Compared to patients treated with PCI, the risk for the primary endpoint decreased with higher plaque burden in patients treated with CABG with HRs of 1.12 (95 %CI 0.70-1.80), 0.82 (95 %CI 0.55-1.20) and 0.75 (95 %CI 0.58-0.94) for CAC<300, CAC 300-1000 and CAC>1000, respectively (Fig. 3 **and Supplementary Figure 3, left panels)**. This was mainly driven by substantially lower risk for spontaneous myocardial infarction during follow-up (HR 0.44, 95 % CI 0.26-0.73), while stroke risk (HR 0.86, 95 % CI 0.52-1.40) and all-cause mortality (HR 0.80, 95 % CI 0.59-1.09) were comparable between PCI and CABG patients (**Supplementary Figure 3, right panels** and **Supplementary Figure 4**). There was no difference in risks within the first 365 days (**Supplementary Figure 5)**.

### 3.3 Adverse outcomes after PCI vs. CABG according to CAC

In patients revascularized with PCI, the risk for atrial fibrillation, heart failure, and bleeding events increased with higher CAC burden (Table 2 **and Supplementary Figure 6**). The association between CAC burden and adverse outcomes in patients revascularized with CABG was less pronounced due to occurrence of procedure-related events across all CAC groups. For patients with CAC <300, the risk of atrial fibrillation (HR 2.32, 95 % CI 1.40-3.82) and heart failure (HR 2.59, 95 % CI 1.24-5.43) was higher in the CABG group compared to the PCI group, mainly due to procedure-related events (**Supplementary Figure 6**). As CAC burden increased, the difference in adverse outcomes between PCI and CABG diminished. Thus, although short-term risk of atrial fibrillation and heart failure was higher in the CABG group for patients with a CAC >1000, long-term risk did not differ due to a high spontaneous occurrence of these events in PCI patients. Bleeding events tended to occur more frequently in PCI patients, especially in those with high CAC scores, driven by higher bleeding events within the first year of revascularization.

**Table 2:** Adverse outcomes defined as atrial fibrillation, heart failure and bleeding events in patients revascularized with percutaneous coronary intervention compared to coronary artery bypass grafting stratified by coronary artery calcification burden.

	Percutaneous coronary intervention (PCI)		Coronary artery bypass grafting (CABG)		Hazard ratio
**Overall**	n	Event rate per 1000 person years	n	Event rate per 1000 person years	
Atrial fibrillation	174	25.6 (22.1-29.7)	209	33.8 (29.5-38.7)	1.31 (1.07-1.60)
Heart Failure	84	11.9 (9.6-14.8)	102	14.9 (12-3-18.1)	1.25 (0.93-1.66)
Bleeding	196	28.4 (24.7-32.6)	144	20.1 (17.0-23.6)	0.71 (0.6-0.9)
Cerebral	26	3.8 (2.6-5.5)	26	3.6 (2.5-5.3)	0.96 (0.56-1.66)
Gastrointestinal	42	6.1 (4.5-8.2)	36	5.0 (3.6-7.0)	0.84 (0.53-1.30)
Urogenital	77	11.1 (8.9-13.9)	49	6.8 (5.2-9.0)	0.62 (0.43-0.88)
Peripheral artery disease	91	13.0 (10.6-16.0)	100	14.3 (11.7-17.3)	1.10 (0.83-1.47)
**CAC<300**					
Atrial fibrillation	23	12.0 (7.9-18.0)	47	28.4 (21.3-37.8)	2.32 (1.40-3.82)
Heart Failure	10	5.0 (2.7-9.4)	24	13.2 (8.9-19.8)	2.59 (1.24-5.43)
Bleeding	41	21.3 (15.9-28.9)	27	14.6 (10.0-21.2)	0.68 (0.41-1.11)
Cerebral	3	1.6 (0.5-4.8)	7	3.8 (1.8-7.9)	2.48 (0.64-9.61)
Gastrointestinal	5	2.6 (1.1-6.2)	5	2.7 (1.1-6.5)	1.00 (0.29-3.48)
Urogenital	21	10.9 (7.1-16.7)	7	3.8 (1.8-7.9)	0.35 (0.15-0.81)
Peripheral artery disease	7	3.5 (1.7-7.3)	10	6.4 (2.9-10.1)	1.06 (0.75-1.50)
**CAC 300-1000**					
Atrial fibrillation	45	22.8 (17.0-30.1)	55	30.6 (23.5-39.9)	1.35 (0.91-2.00)
Heart Failure	14	6.8 (4.0-11.5)	20	9.9 (6.4-15.3)	1.46 (0.73-2.89)
Bleeding	48	24.2 (18.3-32.2)	41	19.4 (14.3-26.4)	0.83 (0.54-1.26)
Cerebral	7	3.5 (1.7-7.4)	5	2.4 (0.9-5.7)	0.70 (0.22-2.18)
Gastrointestinal	9	4.5 (2.4-8.7)	9	4.3 (2.2-8.2)	0.96 (0.38-2.43)
Urogenital	21	10.6 (6.9-16.3)	19	9.0 (5.7-14.1)	0.88 (0.47-1.64)
Peripheral artery disease	21	10.3 (6.7-15.9)	22	10.5 (6.9-16.0)	1.02 (0.56-1.86)
**CAC >1000**					
Atrial fibrillation	106	36.5 (30.1-44.2)	107	39.0 (32.3-47.1)	1.06 (0.81-139)
Heart Failure	60	20.1 (15.6-25.9)	58	19.3 (14.9-25.0)	0.96 (0.67-1.38)
Bleeding	107	35.6 (29.5-43.1)	76	23.7 (18.9-29.7)	0.67 (0.50-0.90)
Cerebral	16	5.3 (3.3-8.7)	14	4.4 (2.6-7.4)	0.81 (0.39-1.67)
Gastrointestinal	28	9.3 (6.4-13.5)	22	6.9 (4.5-10.4)	0.75 (0.43-1.30)
Urogenital	35	11.7 (8.4-16.2)	23	7.2 (4.8-10.8)	0.62 (0.37-1.05)
Peripheral artery disease	63	21.4 (16.7-27.3)	68	22.0 (17.4-18.0)	1.12 (0.56-1.89)

### 3.4 Sensitivity analyses

In sensitivity analyses, we assessed the risk for PAD that is not expected to be affected by revascularization procedure in patients with similar prevalence of risk factors and plaque burden. As shown in Table 2 and **Supplementary Figure 7** the occurrence of PAD was similar in patients undergoing CABG compared to PCI with HR’s of 1.06 (95 % CI 0.75-1.50), 1.02 (95 % CI 0.56-1.86) and 1.12 (95 % CI 0.56-1.89) for CAC<300, CAC 300-1000 and CAC>1000.

In another sensitivity analyses, we compared the risk in those undergoing PCI to patients with CCTA-defined obstructive CAD but who were not revascularized (untreated stenoses). As shown in **Supplementary Figure 8**, the cumulative risk in those with obstructive CAD that did not undergo revascularization were comparable to that of patients with obstructive CAD that underwent PCI. Thus, the risk for the primary endpoint across the 3 CAC strata were comparable for patients undergoing PCI and those with obstructive CAD that did not undergo revascularization, while patients undergoing CABG had lower risk at high CAC>1000 (**Supplementary Figure 9**).

Finally, we re-analyzed the data including the entire population (without matching) adjusting for baseline characteristics in Cox proportional hazard models. As shown in **Supplementary Table 7**, the results remained similar the main analyses.

## 4 Discussion

This study, involving 85,512 patients without known ischemic heart disease undergoing CCTA for symptoms suggestive of CAD, provides novel insights into the role of coronary plaque burden as a determinant of the potential long-term benefit from revascularization with CABG vs. PCI. Several key observations from our analyses help elucidate the mechanism behind the well-documented, yet poorly understood, benefit of CABG over PCI in patients with multivessel obstructive CAD. First, coronary plaque burden, assessed by CAC, had a stronger impact on long-term risk in patients with non-obstructive CAD and patients treated with PCI compared to CABG patients. Notably, this was explained by CAC burden not being associated with myocardial infarction risk after CABG, indicating that CABG diminished the effect of high plaque burden on coronary events. Second, patients with non-obstructive CAD and those treated with PCI had similar long-term risks for the primary endpoint when stratified by plaque burden. This finding shows that targeting obstructive plaques through PCI does not reduce the risk associated with an overall high plaque burden. Third, for patients with low plaque burden, the risk was low regardless of whether patients had non-obstructive CAD or had obstructive CAD being revascularized with PCI or CABG. However, as plaque burden increased, CABG was associated with lower long-term risk than both patients with non-obstructive CAD and patients revascularized with PCI, despite a much higher prevalence of multivessel obstructive CAD. This lower risk was nearly exclusively due to a substantial lower risk for spontaneous myocardial infarctions. Taken together, the totality of these findings suggests that the observed long-term benefit with CABG vs. PCI in patients with multivessel obstructive CAD is not solely attributable to treatment of obstructive plaques. Rather, it is likely mediated by CABG providing more comprehensive revascularization of coronary plaques, encompassing both obstructive and non-obstructive lesions, resulting in a much lower myocardial infarction risk.

The optimal revascularization strategy and its long-term benefits in patients with stable CAD remain a topic of debate [25]. While the role of coronary plaque burden as a significant driver of future cardiovascular events is well recognized in patients without known CAD, its prognostic role in patients with obstructive CAD is still not fully appreciated [[26], [27], [28], [29], [30]]. Traditionally, risk assessment and treatment decisions in patients with obstructive CAD have focused on characteristics of obstructive lesions identified through coronary angiography, such as the number and location of lesions (e.g., using SYNTAX score) [31]. Early studies supported the notion that patients with multiple stenoses had a higher risk of events compared to those with no or fewer stenoses. However, randomized controlled trials like COURAGE, FAME-II, and ISCHEMIA have raised doubts regarding whether the observed high risk can solely be attributed to the presence of obstructive plaques, as they have not shown significant risk reduction with PCI [4,6,7]. Additionally, observational studies highlight a strong correlation between overall plaque burden and the presence of obstructive CAD, with high plaque burden also associated with a higher prevalence of multi-vessel obstructive CAD [29]. Thus, the increased risk observed in patients with obstructive CAD and/or multi-vessel obstructive CAD may instead be due to the higher overall plaque burden in these patients. Our findings provide new data to support this interpretation by demonstrating that long-term risk in patients with obstructive CAD undergoing PCI is similar to that observed in patients with non-obstructive CAD when stratified by plaque burden. Thus, patients with extensive non-obstructive CAD (i.e., CAC >1000) have a substantially higher risk than patients with obstructive CAD but less plaque burden (i.e., CAC <300). This clearly demonstrates that risk is better reflected by plaque burden than by the presence of obstructive plaques, and should facilitate the implementation of intensive preventive medications in patients with high plaque burden, regardless of the presence of stenoses.

Several randomized trials confirm the long-term benefits of CABG over PCI, particularly in patients with three-vessel disease. For example, the SYNTAX study's 5-year outcome results demonstrated a significant 50 % reduction in myocardial infarction with CABG, while the effects on all-cause mortality and stroke were not statistically significant [32]. Likewise, two recent large observational studies have suggested improved prognosis with CABG vs. PCI in patients with heart failure and non-ST elevation myocardial infarction [12,13]. The mechanism underlying the substantially improved coronary prognosis with CABG has been a subject of long debate. Our findings suggest that the benefit is not attributed to bypassing obstructive plaques, but rather to the extensive revascularization of both obstructive and non-obstructive coronary plaques. Specifically, our analyses reveal that in patients with low plaque burden, the risk of future events is low regardless of the number of coronary stenoses or revascularization strategy. However, in patients with high plaque burden, CABG is associated with a better long-term prognosis compared to PCI, primarily due to a significantly lower occurrence of myocardial infarctions. More striking, among patients with CAC>1000, those who underwent CABG for obstructive CAD had a lower risk of long-term events than patients with non-obstructive CAD who were not revascularized. These findings suggest that the benefit of CABG is largely independent of obstructive plagues, and are consistent with previous studies showing that ischemia detection is insufficient in predicting the benefit of CABG or PCI. Interestingly, in the SYNTAX trial, the SYNTAX score, which integrates the severity of CAD based on the number and anatomical characteristics of obstructive lesions, was a strong predictor of outcomes in patients with three-vessel obstructive CAD undergoing PCI, but not in those undergoing CABG [32]. Therefore, low SYNTAX scores (<22) did not show a difference in risk between CABG and PCI, whereas CABG was associated with significantly lower risk at high SYNTAX scores (>33). While the exact reason for the prognostic value of the SYNTAX score is unclear, a likely explanation based on our results is that it may simply serve as an angiography-based measure of overall plaque burden.

Causality between severity of coronary calcification and benefit of CABG vs. PCI cannot be claimed from the findings in this observational study. Therefore, we do not recommend making decisions on revascularization strategy in patients with obstructive CAD based on a specific CAC threshold. Instead, we propose that future randomized trials investigate CABG vs. PCI benefits while taking into account total plaque burden, as it is a strong determinant of long-term risk regardless of number of obstructive plaques. In western Denmark, CCTA has been the preferred first-line test strategy for patients with stable chest pain for over a decade. This testing strategy has recently received a 1A recommendation in the latest US societal guidelines for the diagnosis of patients with stable chest pain [33]. Additionally, CCTA and CT-derived measures of coronary physiology can aid in planning revascularization procedures by assessing disease complexity, vessel size, lesion length, and identifying optimal landing zones for stents and grafts [[34], [35], [36]]. Consequently, it is expected that CCTA and information on plaque burden will be available for the majority of patients scheduled for revascularization in the future.

### 4.1 Strength and limitation

The study has several limitations. Firstly, due to its observational nature, the findings are susceptible to potential selection bias and residual confounding. Secondly, we were unable to assess the appropriateness of the chosen revascularization strategy or identify any individual or local barriers that may have influenced the decision-making process or the utilization of the heart team approach. Additionally, information regarding coronary complexity beyond per-patient CAC and stenosis, such as the SYNTAX score, as well as surgical risk assessments like STS or EuroSCORE, was not available for analysis.

Despite these limitations, the study also has notable strengths. First, we were able to leverage a very large cohort of patients who underwent guideline-directed CCTA as the initial diagnostic test for stable chest pain evaluation. This is particularly valuable given the relatively small proportion of patients with symptoms suggestive of CAD who ultimately undergo CABG or PCI. Thus, a very large cohort like ours was needed to conduct the study effectively. Equally important is the fact that the Danish National Health Service provides universal and comprehensive tax-supported healthcare to all citizens, and physicians' reimbursement is not influenced by whether patients undergo revascularization or the specific mode of revascularization strategy employed. This likely reduces the risk of inappropriate revascularization choices and adds to the strength of the study design. Further, the overall results are in line with general results from previous RCTs demonstrating that CABG primarily improve coronary outcomes as compared to PCI. Finally, the results were robust in sensitivity analyses. Explaining our results through residual confounding is challenging. This would require the confounder to be primarily associated with a reduced risk of spontaneous myocardial infarctions while exerting minimal effects on other atherosclerotic cardiovascular endpoints, such as peripheral artery disease and stroke. The existence of such a confounder selectively associated with myocardial infarction seems implausible.

## 5 Conclusion

Among stable CAD patients with high, but not low, coronary plaque burden as assessed by CAC, revascularization with CABG was associated with lower long-term risk than revascularization with PCI, despite a notably higher prevalence of multivessel obstructive CAD. This better outcome was predominantly driven by a substantial lower occurrence of myocardial infarction. Taken together, these findings suggest that the well-documented benefit of CABG over PCI in patients with extensive CAD are, at least partly, attributed to its ability to bypass a greater plaque burden including non-obstructive lesions in addition to the targeted obstructive lesions. The present study highlights the need for randomized trials on the benefit of revascularization with CABG vs. PCI taking into account the total and vessel specific coronary plaque burden.

## Funding

This study was funded by Aarhus University Hospital.

## Disclosures

Martin Bødtker Mortensen reports lecture fees from Novo Nordisk, Novartis, Sanofi, AstraZeneca and Amarin. Michael Blaha reports grants from the National Institutes of Health, US Food and Drug Administration, American Heart Association, and Aetna Foundation; grants and personal fees from Amgen; and personal fees from Sanofi, Regeneron, Novartis, Bayer, and Novo Nordisk outside the submitted work. The other authors have reported that they have no relationships relevant to the contents of this paper to disclose.

## CRediT authorship contribution statement

**Martin Bødtker Mortensen:** Conceptualization, Formal analysis, Investigation, Methodology, Project administration, Resources, Writing – original draft. **Jesper Møller Jensen:** Data curation, Methodology, Project administration, Writing – review & editing. **Hans Erik Bøtker:** Data curation, Methodology, Writing – review & editing. **Michael Maeng:** Data curation, Methodology, Writing – review & editing. **Kevin Olesen:** Data curation, Project administration, Writing – review & editing. **Mariann Tang:** Methodology, Writing – review & editing. **Niels Peter Rønnow Sand:** Methodology, Writing – review & editing. **Erik Grove:** Methodology, Writing – review & editing. **Kristian Kragholm:** Data curation, Writing – review & editing. **Lars Lyhne Knudsen:** Data curation, Writing – review & editing. **Martin Busk:** Data curation, Writing – review & editing. **Kristian Øvrehus:** Data curation, Writing – review & editing. **Michael J. Blaha:** Conceptualization, Writing – review & editing. **Patrick Serruys:** Methodology, Writing – review & editing. **Henrik Toft Sørensen:** Methodology, Writing – review & editing. **Bjarne Linde Nørgaard:** Conceptualization, Data curation, Methodology, Project administration, Supervision, Writing – review & editing.

## Declaration of competing interest

The authors declare the following financial interests/personal relationships which may be considered as potential competing interests:

Martin Mortensen reports a relationship with Novo Nordisk that includes: speaking and lecture fees. Martin Mortensen reports a relationship with Novartis that includes: speaking and lecture fees. Martin Bødtker Mortensen reports lecture fees from Novo Nordisk, Novartis, Sanofi, AstraZeneca and Amarin. Michael Blaha reports grants from the National Institutes of Health, US Food and Drug Administration, American Heart Association, and Aetna Foundation; grants and personal fees from Amgen; and personal fees from Sanofi, Regeneron, Novartis, Bayer, and Novo Nordisk outside the submitted work. The other authors have reported that they have no relationships relevant to the contents of this paper to disclose. If there are other authors, they declare that they have no known competing financial interests or personal relationships that could have appeared to influence the work reported in this paper.

## Data Availability

The data underlying this article will be shared on reasonable request to the corresponding author.

## Appendix Supplementary materials

Image, application 1
