# Supplementary material for: Coronary plaque burden as a determinant of cardiovascular outcomes in patients undergoing percutaneous coronary intervention versus coronary artery bypass grafting: The Western Denmark Heart Registry
Source: Am J Prev Cardiol. 2026 Feb 18;26:101429. doi: 10.1016/j.ajpc.2026.101429 (PMC13084121; doi:10.1016/j.ajpc.2026.101429)
Supplement: Supplementary file 1 [file mmc1.docx]

**Supplementary Materials**

**Coronary atherosclerotic plaque burden as a determinant of cardiovascular outcomes in patients undergoing percutaneous coronary intervention versus coronary artery bypass grafting:**

**The Western Denmark Heart Registry**

Martin Bødtker Mortensen MD PhD^1,2^, Jesper Møller Jensen MD PhD^1^ , Hans Erik Bøtker MD DMSc^1^, Michael Maeng MD PhD^1^, Kevin Olesen MD PhD^1^, Mariann Tang MD PhD^3^, Niels Peter Rønnow Sand MD PhD^4^, Erik Grove MD PhD^1^, Kristian Kragholm MD PhD^5^, Lars Lyhne MD PhD^6^, Martin Busk MD PhD^7^, Kristian Øvrehus MD PhD^8^, Michael J. Blaha MD MPH^2^, Patrick Serruys MD PhD^9^, Henrik Toft Sørensen MD DMSc^10^, Jagat Narula MD^11^, Bjarne Linde Nørgaard MD DMSc^1^

**Supplementary Table 1. Comorbidity coding**

| **Comorbidity** | **ICD-8** | **ICD-10** |
| --- | --- | --- |
| Myocardial infarction | 410 | I21, I22, I23 |
| Congestive heart failure | 427.09; 427.10; 427.11; 427.19; 428.99; 782.49 | I50; I11.0; I13.0; I13.2 |
| Peripheral vascular disease | 440; 441; 442; 443; 444; 445 | I70; I71; I72; I73; I74; I77 |
| Cerebrovascular disease | 430-438 | I60-I69; G45; G46 |
| Dementia | 290.09-290.19; 293.09 | F00-F03; F05.1; G30 |
| Chronic pulmonary disease | 490-493; 515-518 | J40-J47; J60-J67; J68.4; J70.1; J70.3; J84.1; J92.0; J96.1; J98.2; J98.3 |
| Connective tissue disease | 712; 716; 734; 446; 135.99 | M05; M06; M08; M09; M30; M31; M32; M33; M34; M35; M36; D86 |
| Peptic ulcer | 530.91; 530.98; 531-534 | K22.1; K25-K28 |
| Mild liver disease | 571; 573.01; 573.04 | B18; K70.0-K70.3; K70.9; K71; K73; K74; K76.0 |
| Diabetes without end organ damage | 249.00; 249.06; 249.07; 249.09; 250.00; 250.06; 250.07; 250.09 | E10.0, E10.1; E10.9; E11.0; E11.1; E11.9 |
| Diabetes with end organ damage | 249.01-249.05; 249.08; 250.01- 250.05; 250.08 | E10.2-E10.8, E11.2-E11.8 |
| Hemiplegia | 344 | G81; G82 |
| Moderate to severe renal disease | 403; 404; 580-583; 584; 590.09; 593.19; 753.10-753.19; 792 | I12; I13; N00-N05; N07; N11; N14; N17-N19; Q61 |
| Non-metastatic solid tumor | 140-194 | C00-C75 |
| Leukaemia | 204-207 | C91-C95 |
| Lymphoma | 200-203; 275.59 | C81-C85; C88; C90; C96 |
| Moderate to severe liver disease | 070.00; 070.02; 070.04; 070.06; 070.08; 573.00; 456.00-456.09 | B15.0; B16.0; B16.2; B19.0; K70.4; K72; K76.6; I85 |
| Metastatic cancer | 195-198; 199 | C76-C80 |
| AIDS | 079.83 | B21-B24 |

ICD-8 and ICD-10 codes: *International Classification of Diseases, Eighth and Tenth Resivions*.

**Supplementary Table 2. Baseline characteristics of The Western Denmark Heart Registry patients included in the study.**

|  | |  |  | |
| --- | --- | --- | --- | --- |
| **Characteristics** | **All** | **CABG** | | **PCI** |
| Participants, n | 85 512 | 1479 | | 4942 |
| Age, mean (years)  Current smoking, % | 58 (50-66)  20 | 65 (58-71)  18 | | 62 (54-70)  17 |
| Male (%) | 48 | 81 | | 35 |
| Plasma parameters, median (mmol/l), IQR |  |  | |  |
| Total cholesterol | 5.1 (4.4-5.8) | 5.1 (4.4-5.8) | | 5.0 (4.2-5.7) |
| LDL cholesterol | 3.0 (2.3-3.7) | 3.0 (2.3-3.7) | | 2.7 (2.1-3.4) |
| HDL cholesterol | 1.4 (1.1-1.7) | 1.4 (1.1-1.7) | | 1.5 (1.2-1.8) |
| Hypertension (%) | 40 | 53 | | 48 |
| Diabetes (%) | 7 | 12 | | 8 |
| Body Mass Index,  median, IQR  Comorbidity level^A^ (%)  None  Low  Moderate  Severe | 26.5 (23.8-29.7)  65  18  17 | 26.8 (24.5-29.4)  57  21  11  12 | | 26.2 (23.5-30.0)  48  20  12  13 |
| CAC score (median,  IQR)  Statin use^B^, %  Aspirin use^C^, % | 3 (0-115)  8  9 | 900 (314-1870)  8  9 | | 343 (90-890)  12  13 |

Data are n (%), median (IQR), or n.

^A^ Levels of comorbidity were based on Charlson Comorbidity Index scores of 0 (low), 1 (moderate), and $\geq$2 (severe).

^B^ Defined as a statin prescription redeemed >180 days before CCTA.

^C^ Defined as an aspirin prescriptions redeemed >180 days before CCTA.

CABG: Coronary artery bypass grafting; PCI: Percutaneous coronary intervention

**Supplementary Table 3. Baseline characteristics of the 1:1:1 propensity score matched patients with coronary artery calcium score <300 from the Western Denmark Heart Registry included in the study.**

|  | |  |  | | |  |  |
| --- | --- | --- | --- | --- | --- | --- | --- |
| **Characteristics** | **CABG** | | | **Matched PCI** | **Matched non-obstructive CAD without revascularisation*** | | |
| Participants, n | 361 | | | 361 | 361 | | |
| Age, mean (years)  Current smoking, % | 60 (52-66)  2 | | | 60 (52-66)  2 | 60 (52-66)  2 | | |
| Male (%) | 79 | | | 80 | 80 | | |
| Hypertension (%) | 41 | | | 41 | 41 | | |
| Diabetes (%)  Previous stroke (%) | 7  2 | | | 6  2 | 6  2 | | |
| Body Mass Index,  median, IQR  Comorbidity level^A^ (%)  None  Low  Moderate  Severe | 26.8 (24.5-29.4))  68  16  10  6 | | | 27.1 (24.7-29.7)  70  17  8  5 | 27.0 (24.5-30.1)  68  16  10  5 | | |
| CAC (median, IQR)  3-vessel disease**, %  Left main disease***, %    Statin use^B^, %  Aspirin use^C^, % | 112 (26-200)  21  11  5  3 | | | 92 (21-189)  4  2  5  3 | 1 (0-48)  0  0  75  4 | | |

Data are n (%), median (IQR), or n.

^A^ Levels of comorbidity were based on Charlston Comorbidity Index scores of 0 (low), 1 (moderate), and $\geq$2 (severe).

^B^ Defined as a statin prescription redeemed >180 days before CCTA.

^C^ Defined as an aspirin prescriptions redeemed >180 days before CCTA.

*Patients with non-obstructive coronary artery disease and did not undergo revascularization within 180 days of CCTA

**CCTA-defined 3-vessel obstructive coronary artery disease

***CCTA-defined left main disease

**Supplementary Table 4. Baseline characteristics of the 1:1:1 propensity score matched patients with coronary artery calcium score 300-1000 from the Western Denmark Heart Registry included in the study.**

|  | |  |  | | |  |  |
| --- | --- | --- | --- | --- | --- | --- | --- |
| **Characteristics** | **CABG** | | | **Matched PCI** | **Matched non-obstructive CAD without revascularisation*** | | |
| Participants, n | 419 | | | 419 | 419 | | |
| Age, mean (years)  Current smoking, % | 64 (58-70)  18 | | | 65 (59-70)  16 | 65 (59-70)  17 | | |
| Male (%) | 81 | | | 81 | 82 | | |
| Hypertension (%) | 47 | | | 48 | 49 | | |
| Diabetes (%)  Previous stroke (%) | 10  4 | | | 7  4 | 8  4 | | |
| Body Mass Index,  median, IQR  Comorbidity level^A^ (%)  None  Low  Moderate  Severe | 26.7 (24.5-29.3))  62  19  10  9 | | | 27.1 (24.9-29.4)  65  17  9  8 | 26.5 (24.2-29.8)  64  19  9  9 | | |
| CAC (median, IQR)  3-vessel disease**, %  Left main disease***, %    Statin use^B^, %  Aspirin use^C^, % | 592 (441-786)  29  10  7  6 | | | 538 (412-712)  14  3  7  9 | 503 (369-675)  0  0  7  9 | | |

Data are n (%), median (IQR), or n.

^A^ Levels of comorbidity were based on Charlston Comorbidity Index scores of 0 (low), 1 (moderate), and $\geq$2 (severe).

^B^ Defined as a statin prescription redeemed >180 days before CCTA.

^C^ Defined as an aspirin prescriptions redeemed >180 days before CCTA.

*Patients with non-obstructive coronary artery disease and did not undergo revascularization within 180 days of CCTA

**CCTA-defined 3-vessel obstructive coronary artery disease

***CCTA-defined left main disease

**Supplementary Table 5. Baseline characteristics of the 1:1:1 propensity score matched patients with coronary artery calcium score >1000 from the Western Denmark Heart Registry included in the study.**

|  | |  |  | | |  |  |
| --- | --- | --- | --- | --- | --- | --- | --- |
| **Characteristics** | **CABG** | | | **Matched PCI** | **Matched non-obstructive CAD without revascularisation*** | | |
| Participants, n | 699 | | | 699 | 699 | | |
| Age, mean (years)  Current smoking, % | 68 (62-73)  19 | | | 68 (62-73)  17 | 67 (62-72)  19 | | |
| Male (%) | 82 | | | 83 | 83 | | |
| Hypertension (%) | 63 | | | 63 | 61 | | |
| Diabetes (%)  Previous stroke (%) | 17  5 | | | 15  5 | 17  5 | | |
| Body Mass Index,  median, IQR  Comorbidity level^A^ (%)  None  Low  Moderate  Severe | 27.4 (24.9-29.9)  57  21  11  11 | | | 27.4 (24.8-30.3)  59  20  9  12 | 27.1 (24.5-30.6)  58  20  10  11 | | |
| CAC (median, IQR)  3-vessel disease**, %  Left main disease***, %    Statin use^B^, %  Aspirin use^C^, % | 1949 (1437-2844)  52  8  9  9 | | | 1740 (1285-2546)  20  2  9  11 | 1819 (1311-2764)  0  0  9  10 | | |

Data are n (%), median (IQR), or n.

^A^ Levels of comorbidity were based on Charlston Comorbidity Index scores of 0 (low), 1 (moderate), and $\geq$2 (severe).

^B^ Defined as a statin prescription redeemed >180 days before CCTA.

^C^ Defined as an aspirin prescriptions redeemed >180 days before CCTA.

*Patients with non-obstructive coronary artery disease and did not undergo revascularization within 180 days of CCTA

**CCTA-defined 3-vessel obstructive coronary artery disease

***CCTA-defined left main disease

**Supplementary Table 6. Statin and aspirin use after computed tomography angiography of the 1:1:1 propensity score matched patients with coronary artery calcium score >1000 from the Western Denmark Heart Registry included in the study.**

|  | |  |  | | |  |  |
| --- | --- | --- | --- | --- | --- | --- | --- |
| **Characteristics** | **CABG** | | | **Matched PCI** | **Matched non-obstructive CAD without revascularisation*** | | |
| **Overall**  Post-CTA statin use^A^, %  Post-CTA aspirin use^B^, %  **CAC<300**  Post-CTA statin use^A^, %  Post-CTA aspirin use^B^, %  **CAC 300-1000**  Post-CTA statin use^A^, %  Post-CTA aspirin use^B^, %  **CAC >1000**  Post-CTA statin use^A^, %  Post-CTA aspirin use^B^, % | 99  93  99  96  98  92  98  93 | | | 99  97  100  98  98  96  98  97 | 82  64  57  34  91  72  91  74 | | |

^A^Defined as a statin prescription redeemed >180 days after CCTA.

^B^ Defined as an aspirin prescriptions redeemed >180 days after CCTA.

**Supplementary Table 7: Association of coronary artery calcium burden with risk for future events in patients with non-obstructive coronary artery disease and in patients with obstructive coronary artery disease revascularized with percutaneous coronary intervention or coronary artery bypass grafting stratified by coronary artery calcification burden.**

|  | Death, MI or stroke | |  | MI | |  | Stroke | |  | Death | |
| --- | --- | --- | --- | --- | --- | --- | --- | --- | --- | --- | --- |
|  | n | Hazard ratio (95% CI) |  | n | Hazard ratio (95% CI) |  | n | Hazard ratio (95% CI) |  | n | Hazard ratio (95% CI) |
| **Nonobstructive CAD and no revascularization (n=1479)** |  |  |  |  |  |  |  |  |  |  |  |
| CAC<300 | 25 | Ref |  | 3 | Ref |  | 6 | Ref |  | 16 | Ref |
| CAC 300-1000 | 55 | 1.52 (0.92-2.45) |  | 10 | 1.53 (0.38-6.22) |  | 9 | 1.03 (0.36-2.93) |  | 40 | 1.79 (0.98-3.26) |
| CAC >1000 | 157 | 2.72 (1.72-4.32) |  | 31 | 4.02 (1.16-14.00) |  | 33 | 1.97 (0.79-4.97) |  | 116 | 2.71 (1.54-4.76) |
|  |  |  |  |  |  |  |  |  |  |  |  |
| **PCI (n=1479)** |  |  |  |  |  |  |  |  |  |  |  |
| CAC<300 | 37 | Ref |  | 15 | Ref |  | 6 | Ref |  | 13 | Ref |
| CAC 300-1000 | 63 | 1.46 (0.94-2.27) |  | 18 | 1.47 (0.70-3.08) |  | 13 | 1.29 (0.47-3.57) |  | 31 | 2.11 (1.08-4.12) |
| CAC >1000 | 166 | 2.45 (1.57-3.51) |  | 45 | 2.29 (1.16-4.54) |  | 34 | 2.14 (0.86-5.33) |  | 93 | 3.53 (1.89-6.56) |
|  |  |  |  |  |  |  |  |  |  |  |  |
| **CABG (n=1479)** |  |  |  |  |  |  |  |  |  |  |  |
| CAC<300 | 35 | Ref |  | 13 | Ref |  | 9 | Ref |  | 15 | Ref |
| CAC 300-1000 | 48 | 0.88 (0.56-1.37) |  | 5 | 0.31 (0.11-0.93) |  | 25 | 1.79 (0.81-3.93) |  | 28 | 1.20 (0.63-2.29) |
| CAC >1000 | 124 | 1.26 (0.84-1.88) |  | 21 | 0.73 (0.32-1.64) |  | 30 | 1.37 (0.62-3.00) |  | 78 | 1.70 (0.94-3.08) |

**Supplementary Table 8: Risk for the primary endpoint in patients revascularized with percutaneous coronary intervention compared to coronary artery bypass grafting stratified by coronary artery calcification and analyses without propensity score matching but controlled for baseline characteristics in Cox proportional hazard models.**

|  |  | Death, MI or stroke |
| --- | --- | --- |
|  | Number of patients | Hazard ratio (95% CI) |
| **CAC < 300**  PCI  CABG | 2322  361 | Ref  1.10 (0.67-1.80) |
|  |  |  |
| **CAC 300-1000**  PCI  CABG | 1532  419 | Ref  0.80 (0.52-1.17) |
| **CAC >1000**  PCI  CABG | 1088  699 | Ref  0.72 (0.50-0.91) |

CAC: coronary artery calcification

PCI: Percutaneous coronary intervention

CABG: Coronary artery bypass grafting

**Supplementary Figure 1. Occurrence of revascularization with coronary artery bypass grafting and percutaneous coronary intervention after computed tomography angiography.**

**
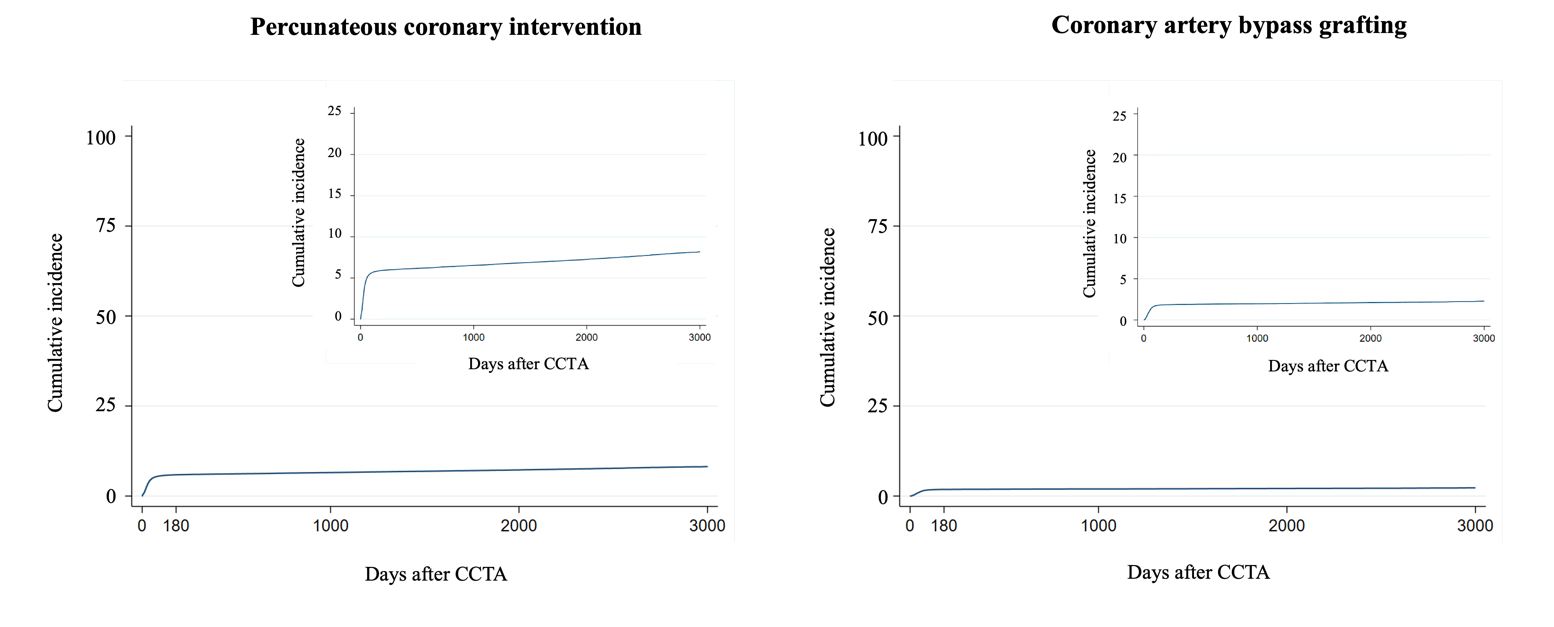
**

**Supplementary Figure 2. Distribution of coronary atery calcification (CAC) in patients undergoing percutaneous coronary intervention and coronary atery bypass grafting within 180 days of computed tomography angiography**


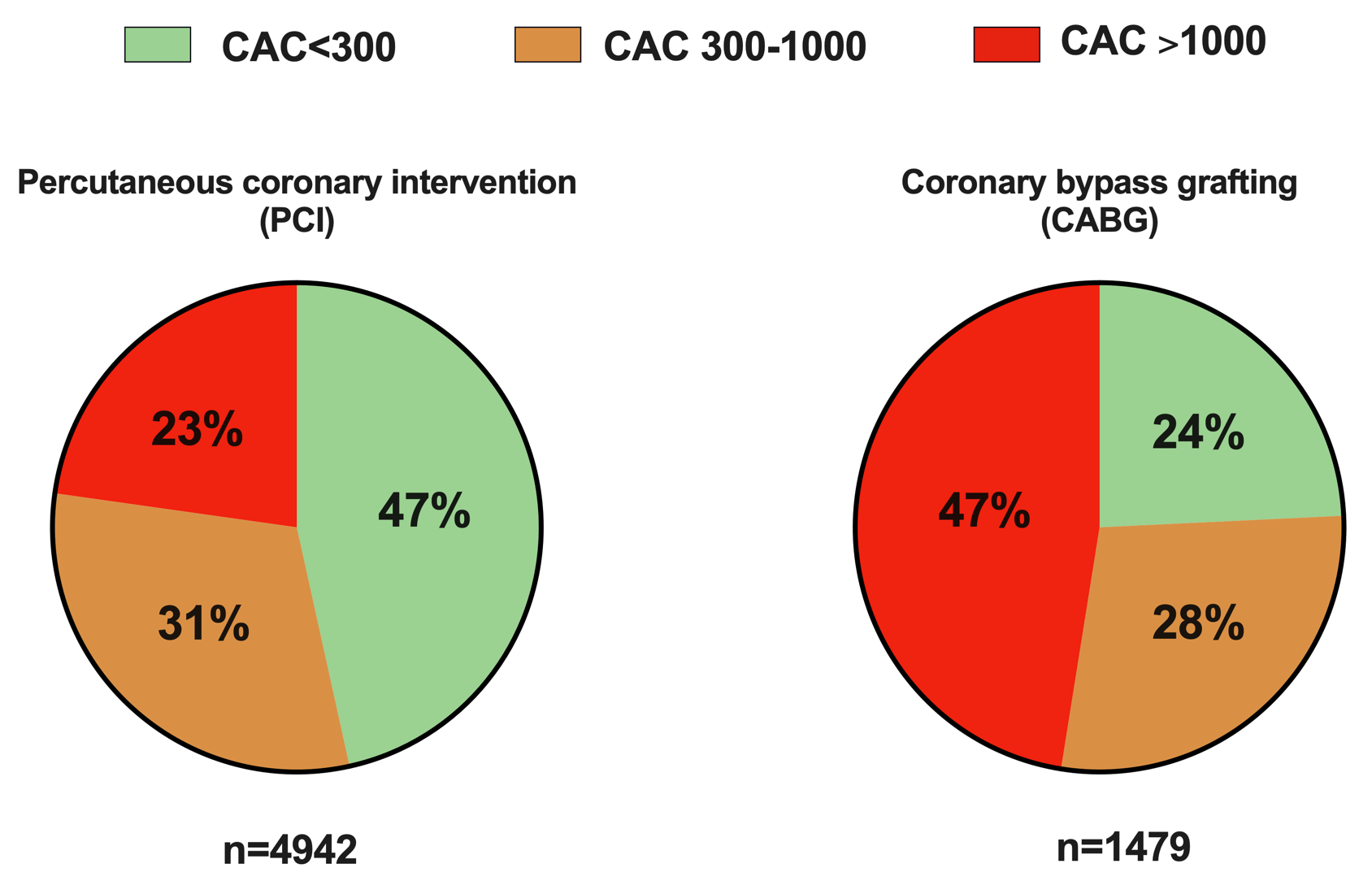


**Supplementary Figure 3. Cumulative incidence curves of myocardial infarction, stroke and all-cause death in patients revascularized with percutaneous coronary intervention compared coronary artery bypass grafting stratified by coronary artery calcification burden.**

**
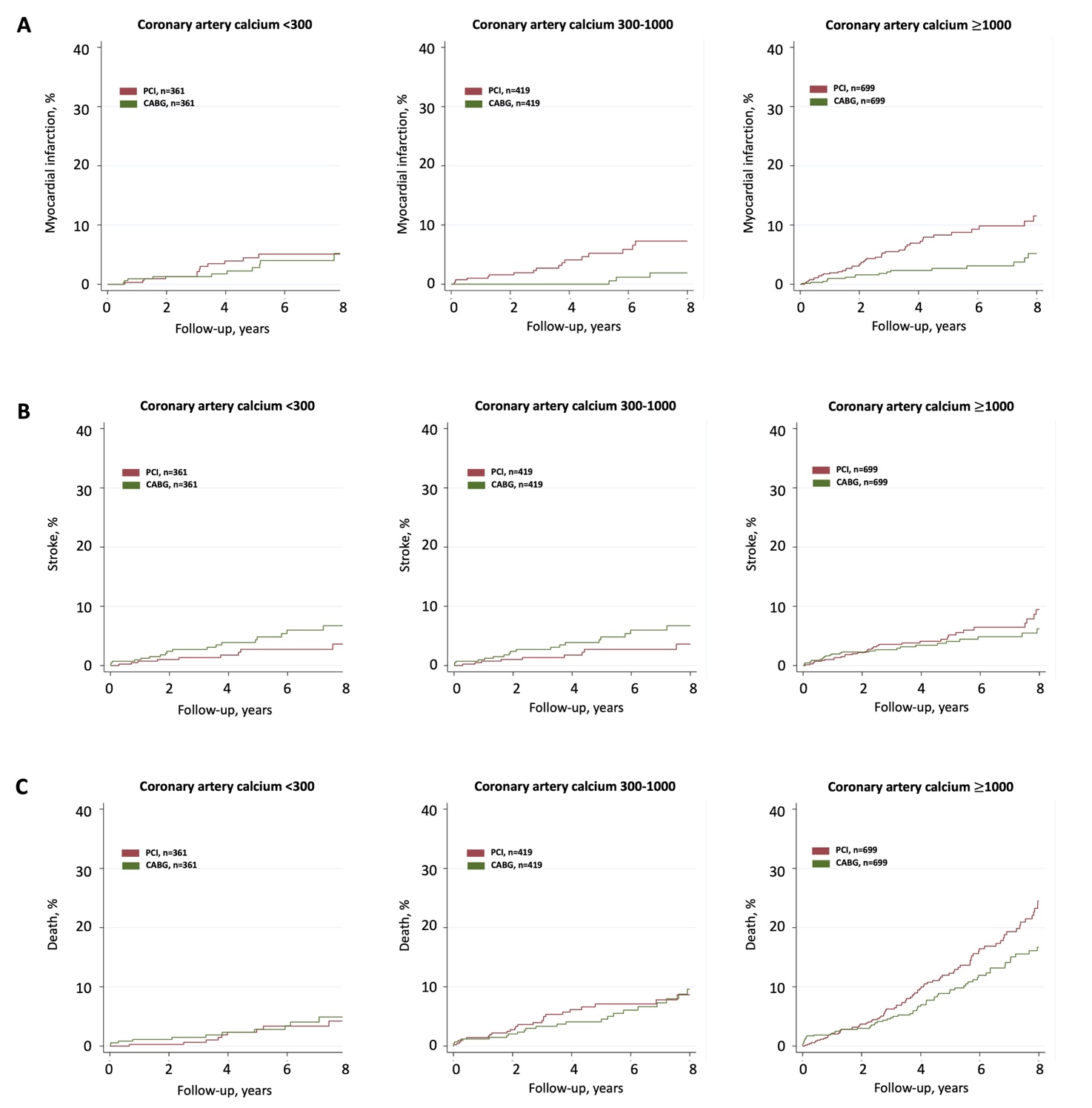
**

**Supplementary Figure 4. Event-rates per 1000 person-years in patients revascularized with percutaneous coronary intervention compared coronary artery bypass grafting stratified by coronary artery calcification burden.**


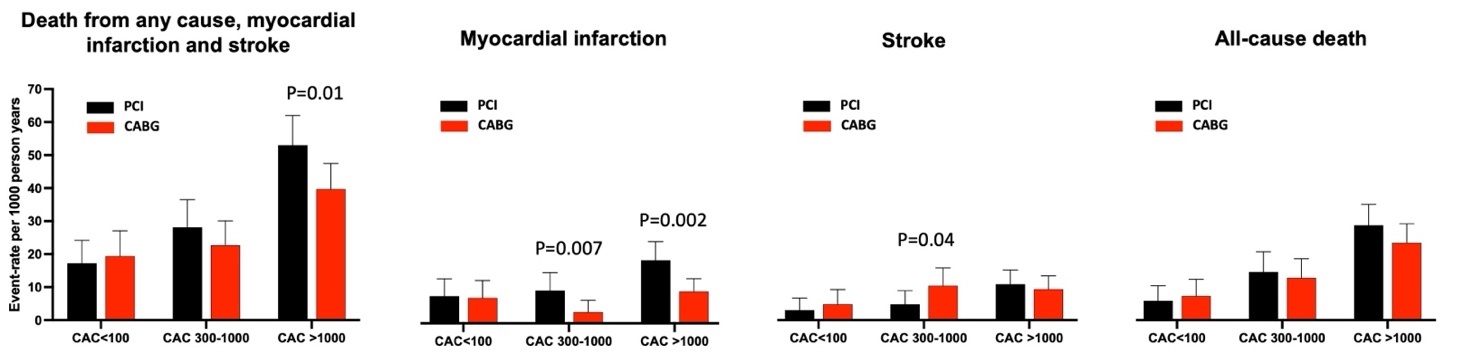


**Supplementary Table 5: Cumulative incidence curves of the primary endpoint occurring within 365 days in patients with non-obstructive coronary artery disease and in patients with obstructive coronary artery disease revascularized with percutaneous coronary intervention or coronary artery bypass grafting stratified by coronary artery calcification burden.**


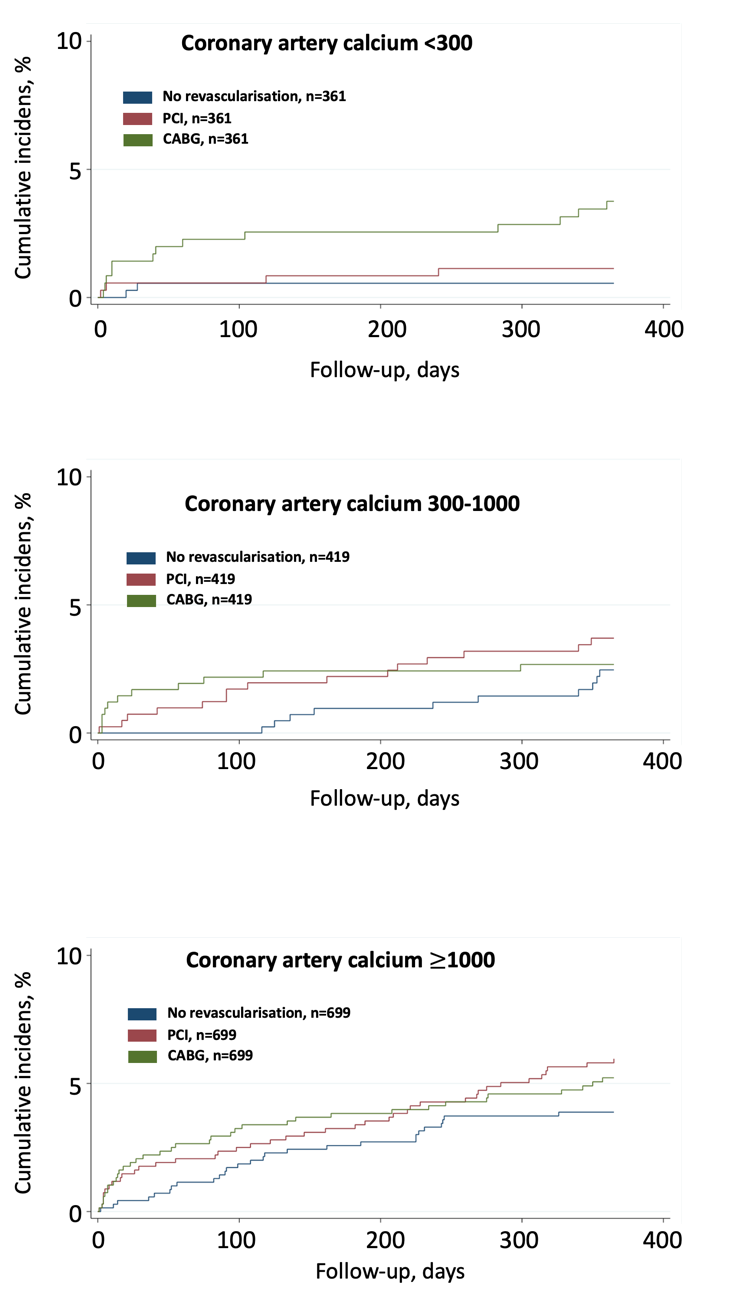


**Supplementary Table 6: Cumulative incidence curves for adverse outcomes defined as atrial fibrillation, heart failure and bleeding events in patients revascularized with percutaneous coronary intervention compared to coronary artery bypass grafting stratified by coronary artery calcification burden.**

**
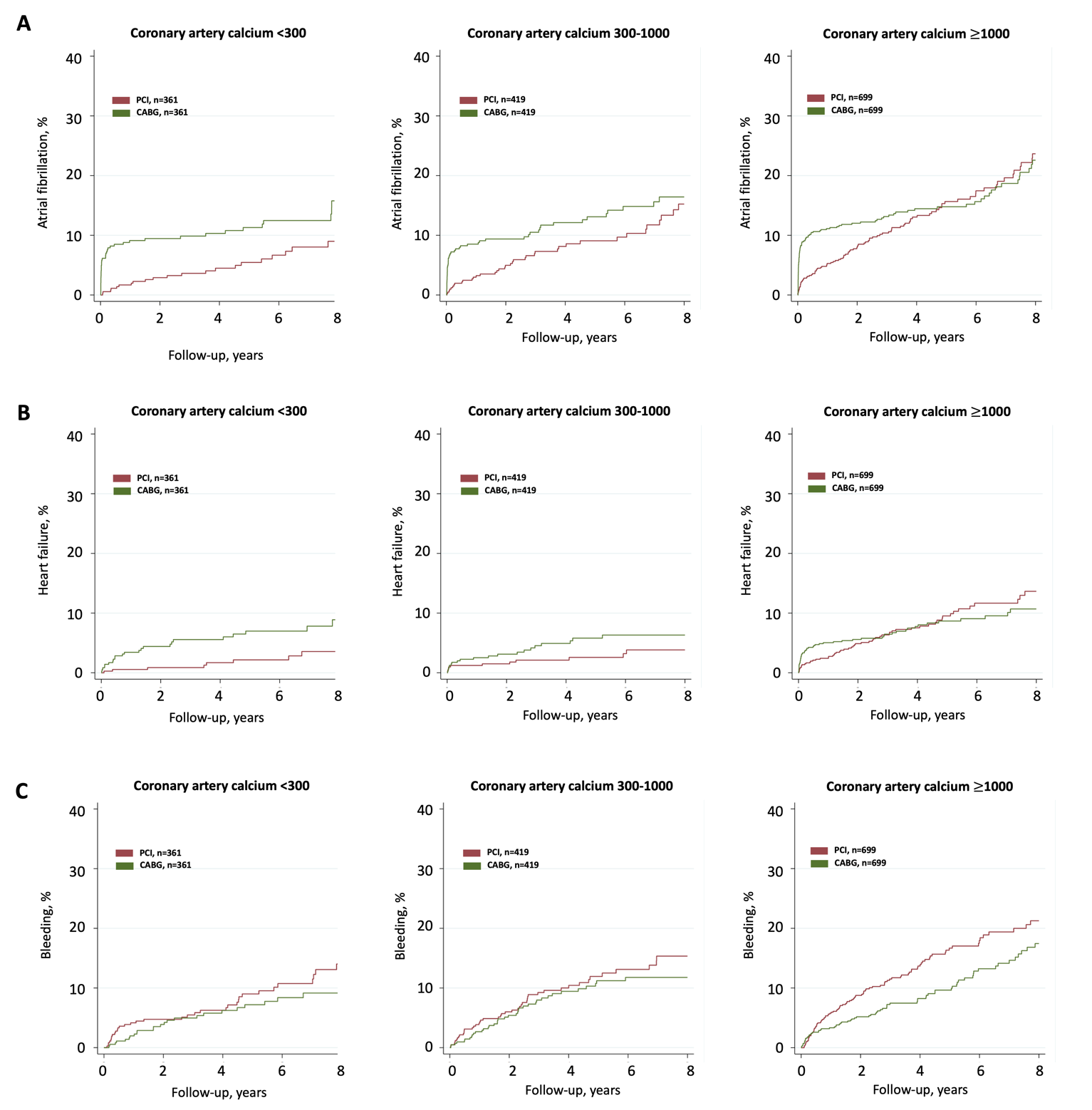
**

**Supplementary Table 7: Cumulative incidence curves for peripheral artery disease in patients revascularized with percutaneous coronary intervention compared to coronary artery bypass grafting stratified by coronary artery calcification burden.**


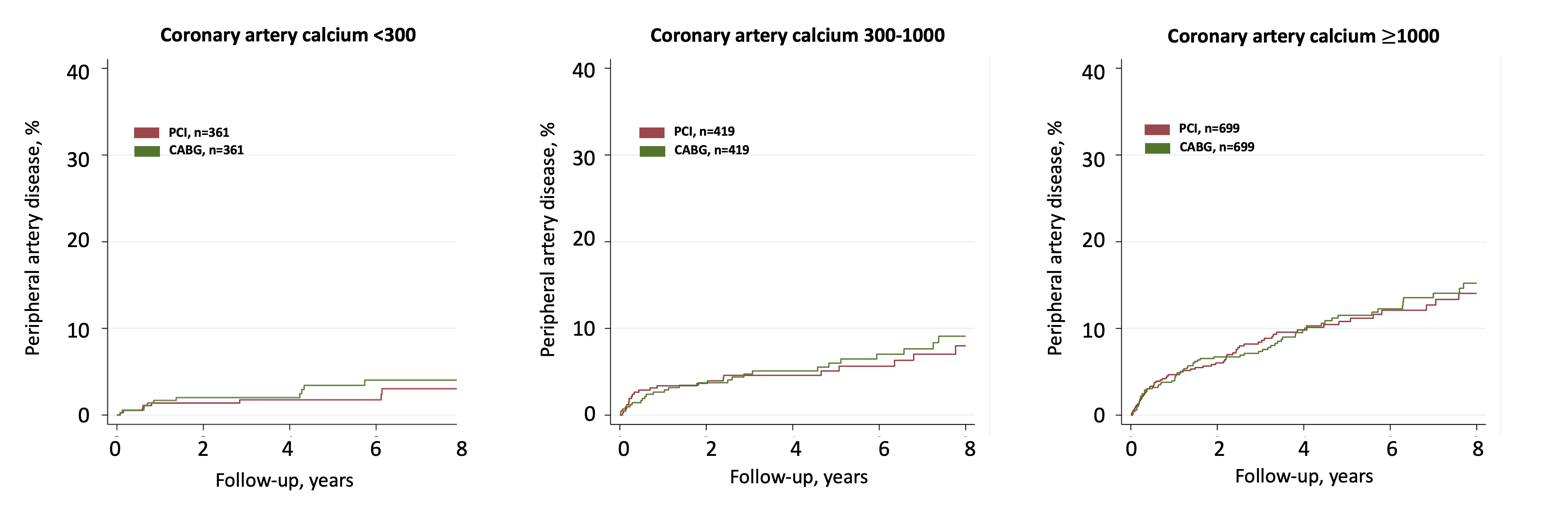


**Supplementary Figure 8. Cumulative incidence curves of the primary endpoint in patients with obstructive coronary artery disease not being revascularized and in patients with obstructive coronary artery disease being revascularized with percutaneous coronary intervention stratified by coronary artery calcification burden.**

**
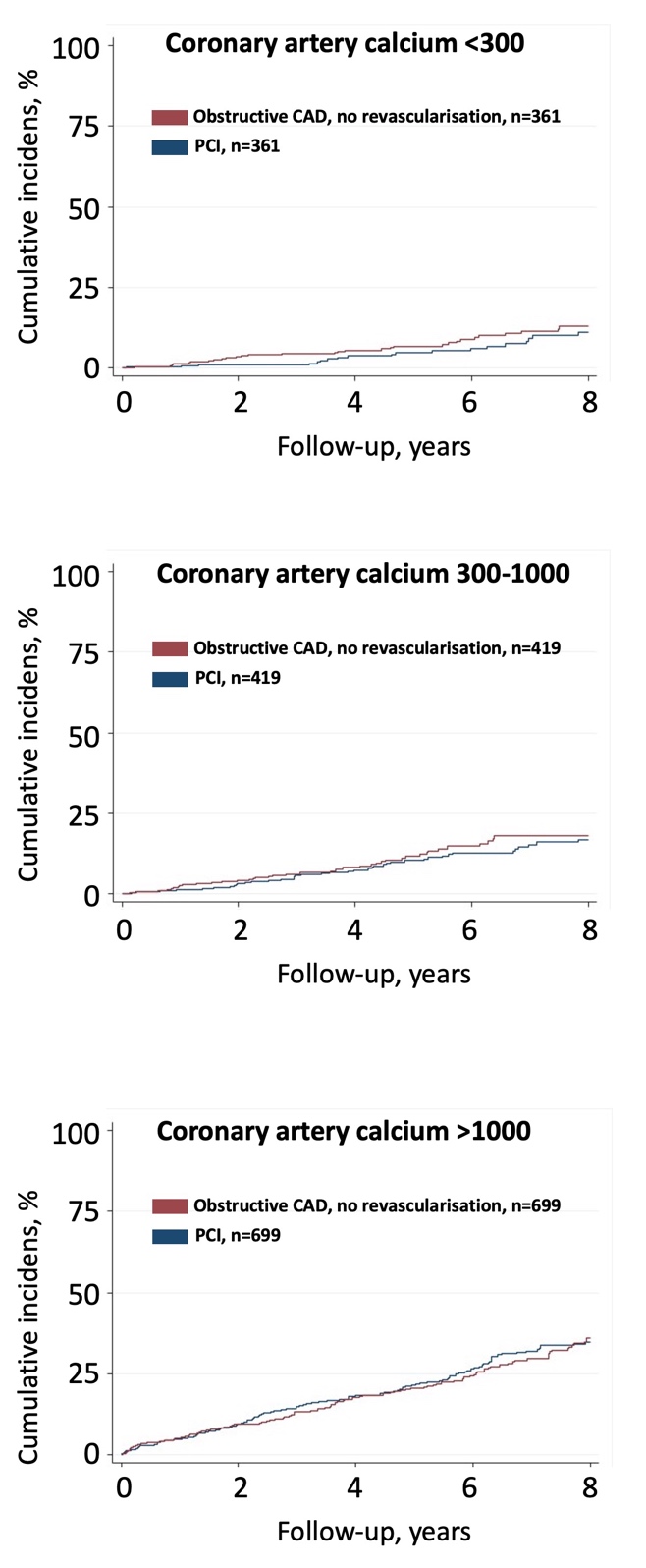
**

**Supplementary Figure 9: Risk for the primary endpoint in patients with obstructive coronary artery disease not being revascularized and in patients with obstructive coronary artery disease revascularized with percutaneous coronary intervention or coronary artery bypass grafting stratified by coronary artery calcification burden.**

**
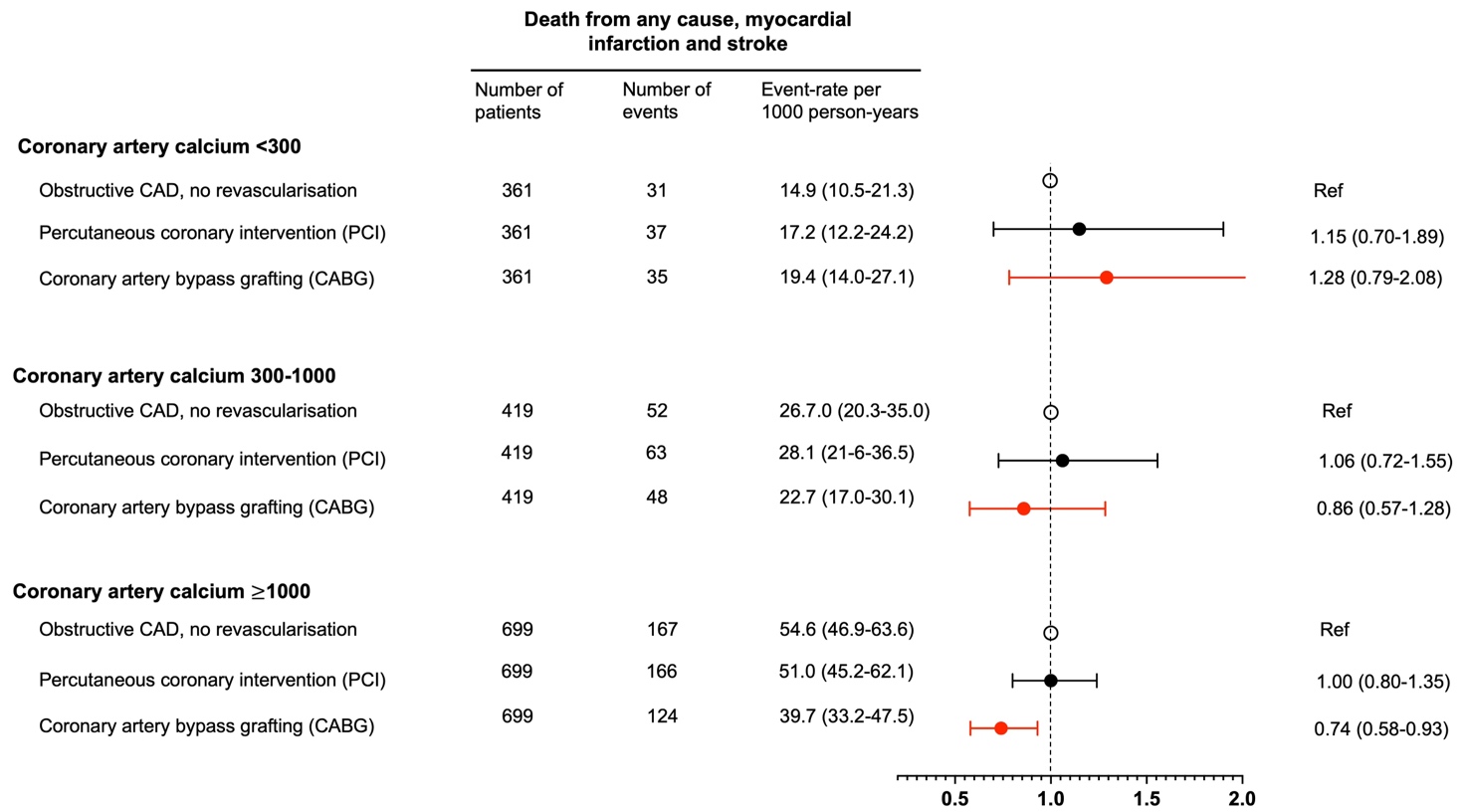
**
